# Management of irritable bowel syndrome in primary care

**DOI:** 10.3399/BJGP.2025.0190

**Published:** 2025-08-25

**Authors:** Sarah L Alderson, Christopher J Black, Alexander C Ford, Hazel A Everitt

**Affiliations:** 1 Leeds Institute of Health Sciences, University of Leeds, Leeds, UK; 2 Leeds Gastroenterology Institute, St James’s University Hospital, Leeds, UK; 3 Leeds Institute of Medical Research, St James’s University Hospital, University of Leeds, Leeds, UK; 4 Primary Care Research Centre, University of Southampton, Southampton, UK

## Introduction

Irritable bowel syndrome (IBS) is a common condition of the gut–brain axis with a population prevalence of 5%.^1^ It follows a chronic fluctuating course. IBS has significant morbidity and socioeconomic impact, with estimated NHS costs of £1.3 to £2 billion per year. Most patients are seen and managed in primary care. Current guideline-recommended treatments improve symptoms in only 30–40% of patients. Thus, many people live with ongoing troublesome IBS symptoms impacting their quality of life, leading to repeated consultations.

Currently, IBS is categorised by predominant stool-type: diarrhoea (IBS-D), constipation (IBS-C), mixed (IBS-M), or unclassified (IBS-U). Most existing recommended treatments have only been assessed in IBS-D and IBS-C sub-types, even though 40% of patients have IBS-M or IBS-U. The National Institute for Health and Care Excellence (NICE) guidance for management of IBS (CG61) was published a decade ago.^1^ Recently published trials have identified effective management options in primary care that should inform updated NICE guidance.

This article summarises the latest evidence for managing IBS in primary care.

## Diagnosis

**Figure 1.**
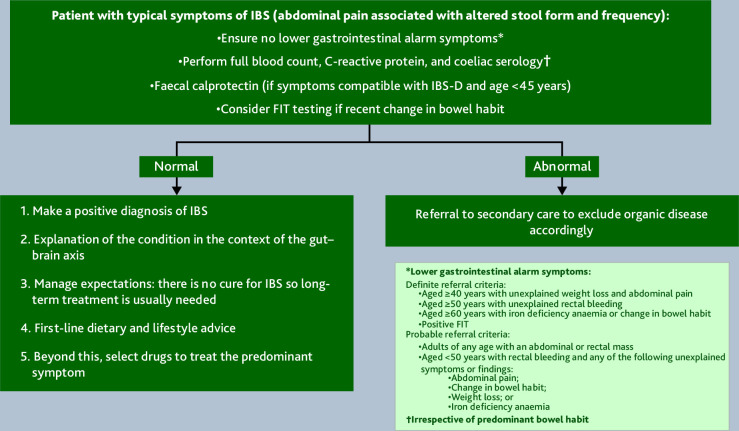
Diagnostic pathway for IBS. D = diarrhoea. FIT = faecal immunochemical test. IBS = irritable bowel syndrome.

IBS is characterised by abdominal pain associated with altered bowel habit and the absence of alarm features. It is not a diagnosis of exclusion, but is made using a positive approach, based on clinical criteria (presence of typical symptoms, and absence of alarm features), and limited investigations (Figure 1). Good communication and a clear explanation of IBS is important to avoid patients feeling ‘dismissed’. Patients have reported ‘negative’ views of healthcare interactions regarding IBS, including feeling unheard and experiencing little empathy.^2^ Clinicians should ask about impact of IBS on social functioning and activities of daily living. Patients may try to manage symptoms by avoiding activities that may exacerbate symptoms (for example, eating out) or cause embarrassment if access to toilets is limited (for example, hiking, travelling). IBS can significantly impact employment if frequent toilet visits are needed. Acknowledging the impact of IBS symptoms on daily life can help patients feel validated, understood, and more able to manage their symptoms.

## Current recommended management

### First-line management

Supporting self-management involves empowering patients to feel more control over their IBS. Patient education and support to develop a greater understanding of their IBS and its impact is key, including understanding their personal triggers such as diet or stress and explaining the pathophysiology of IBS in terms of the gut–brain axis. Dietary advice (including discussion of available diets with known effectiveness),^3^ reduction of stress, regular exercise, and relaxation activities are recommended.^1^ The NICE food fact sheet recommends regular mealtimes and avoiding foods that exacerbate symptoms, including alcohol, artificial sweeteners, and caffeine. Limiting processed foods and ensuring adequate hydration may be beneficial.^3^ Soluble fibre (oats/ispaghula husk) can help constipation, but insoluble fibre (such as bran) may worsen abdominal pain and bloating.

NICE guidance recommends first-line medications based upon IBS subtype, including to slow gut transit for diarrhoea and urgency symptoms (for example, loperamide), laxatives to improve constipation (for example, macrogols), and antispasmodics for abdominal pain and bloating (for example, peppermint oil, hyoscine, etc.). Traditional analgesics (for example, paracetamol, non-steroidal anti-inflammatory drugs, and opioids) are unlikely to improve IBS-related pain and should be avoided.

### Second-line management

The potential benefits of a low FODMAP (fermentable oligosaccharides, disaccharides, monosaccharides, and polyols) diet should be discussed with patients.^3^ Suitable, motivated patients should be referred to a dietician for consideration of this.^1,4^ It should not be attempted without dietetic support because of its complexity and risk of nutritional deficiencies, and should be avoided in those with a history of disordered eating because its restrictive nature may exacerbate this.^3^

NICE guidance suggestions for potential second-line options include tricyclic antidepressants (TCA) or selective serotonin reuptake inhibitors (SSRIs),^1^ which may act as gut–brain neuromodulators in IBS and have effects on pain signalling and gastrointestinal motility; and psychological therapies such as cognitive behavioural therapy (CBT) or hypnotherapy. However, the guidelines highlighted that the evidence was limited, and further research was needed.

## New and emerging management options

Since NICE guidance publication, people’s understanding of IBS and its management has improved.^4^ Two recent large National Institute for Health and Care Research-funded UK randomised controlled trials have evaluated IBS treatments.

The ACTIB trial, which recruited 558 patients, found that IBS-specific cognitive behavioural therapy (CBT) delivered by telephone or internet improved global IBS symptoms, compared with routine care, with ongoing beneficial effects 24 months after treatment completion.^5^ Helping patients manage thoughts, behaviours, emotions, and symptoms related to IBS can reduce its impact. CBT for IBS is now available via NHS talking therapy services. Earlier use of CBT for IBS could be beneficial, improving patient empowerment and understanding of IBS.

**Figure 2.**
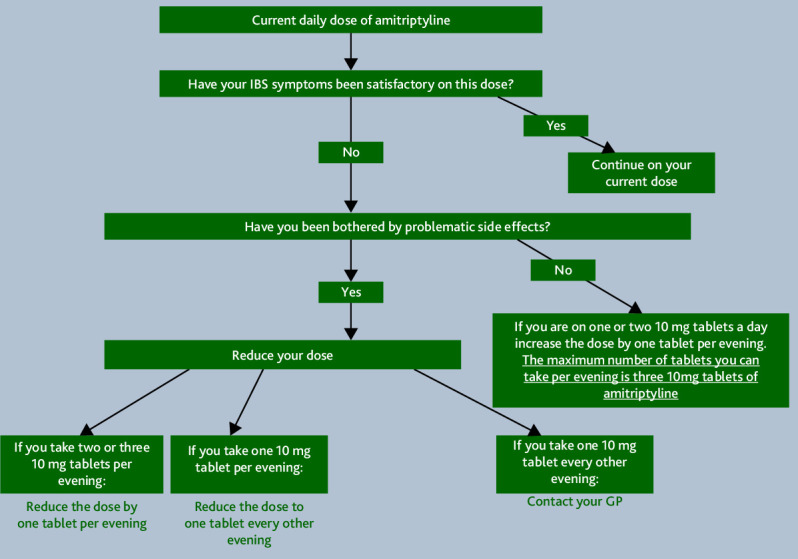
Amitriptyline dose-titration pathway. IBS = irritable bowel syndrome.

ATLANTIS, the largest trial of a tricyclic antidepressant versus placebo for IBS to date with 463 patients,^6^ found that low-dose patient self-titrated (Figure 2) amitriptyline was effective, well-tolerated, and acceptable to patients and GPs,^2^ irrespective of IBS sub-type.^7^ Previously rarely prescribed for IBS in primary care, amitriptyline now has evidence to support more widespread use in IBS. The patient self-dose titration document is freely available to download from the trial website (https://ctru.leeds.ac.uk/atlantis/)

Other IBS treatments show promise, but more research is needed in primary care populations, which differ from those seen in secondary care where patients are more likely to have a severe IBS profile.^4^ The TRITON trial tested titrated ondansetron versus placebo for IBS-D in secondary care.^8^ Although underpowered, when combined with other trials in a meta-analysis, it was found to improve diarrhoea and urgency symptoms. Enterosgel, an intestinal adsorbent, also has some trial evidence for diarrhoea and urgency symptoms. Linaclotide, a guanylate cyclase-C receptor agonist that increases intestinal secretions and reduces visceral pain, has promising results in IBS-C.^9^ Duloxetine is effective for other persistent painful conditions, but data in IBS are lacking. Gut-directed hypnotherapy has evidence indicating effectiveness, but access in primary care is limited, although web-based access might improve this.

## When to refer to secondary care

Most patients with IBS can be successfully managed in primary care. Secondary care referral is indicated where there is diagnostic uncertainty, or if symptoms are severe and refractory to multiple treatments given an adequate trial.

## Summary

Since the NICE guidance for IBS was published, large UK-based research trials have provided evidence of effective treatments for the management of IBS in primary care. Although currently underutilised, these are effective, acceptable to patients and GPs, and available as options for management in primary care. Other IBS treatments show promise but need further research evidence in primary care.
